# Supplementary material for: The effect of horseshoes and surfaces on horse and jockey centre of mass displacements at gallop
Source: PLoS One. 2021 Nov 23;16(11):e0257820. doi: 10.1371/journal.pone.0257820 (PMC8610270; doi:10.1371/journal.pone.0257820)
Supplement: S1 File — (DOCX) [file pone.0257820.s001.docx]

**Supporting Information**

**Supplementary Methods**

**Field site**

An aerial view of the two racetracks utilized in this study is provided below (Fig. S1).


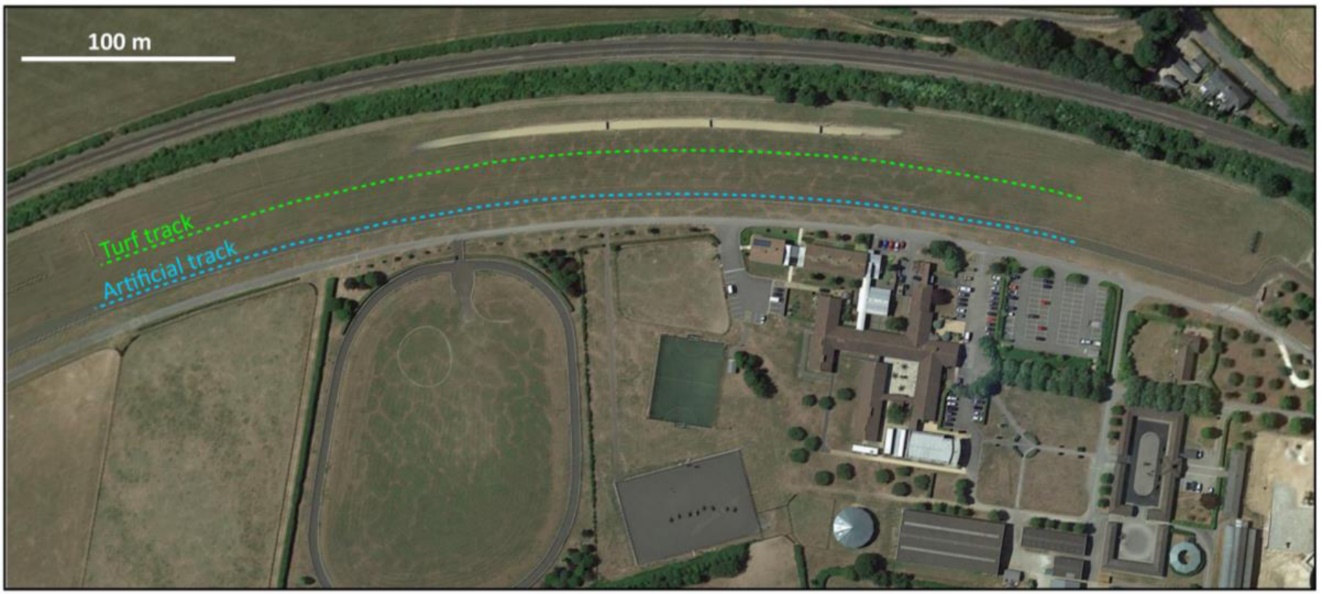


**Fig S1. Aerial photograph of the British Racing School illustrating the approximate paths taken by the horse and rider per gallop run on the turf and artificial tracks.** Image from Google Maps [117].

Table S1 summarises the runs completed for each horse-rider dyad.

**Table S1. Number of gallop runs for each horse-rider combination in the different shoe-surface combinations.**

| **Horse-ID** | **Jockey-ID** | **Aluminium-Artificial** | **Aluminium-Turf** | **Barefoot-Artificial** | **Barefoot-Turf** | **GluShu**  **-Artificial** | **GluShu-**  **Turf** | **Steel-Artificial** | **Steel-**  **Turf** |
| --- | --- | --- | --- | --- | --- | --- | --- | --- | --- |
| 1 | 3 | 2 | 2 | 2 | 2 | 2 | 2 | 3 | 2 |
| 3 | 3 | 4 | 2 | 3 | 3 | 2 | 2 | 4^R^ (2+2) | 4^R^ (2+2) |
| 4 | 3 | 2 | 2 | 2 | 2 | 2 | 3 | 2 | 3 |
| 5 | 3 | 5 | NA | 3 | NA | NA | NA | NA | NA |
| 6 | 3 | 3 | NA | 2 | NA | 2 | 4 | 3 | NA |
| 7 | 3 | 2 | NA | 4^R^ (2+2) | 3 | 2 | NA | 2 | 2 |
| 8 | 4 | 2 | 2 | 3 | 2 | 2 | 4^R^ (2+2) | 2 | 2 |
| 9 | 3 | NA | NA | 2 | NA | 3 | NA | NA | NA |
| 10 | 4 | 4 | 2 | 2 | 2 | 3 | 3 | 4 | 2 |
| 11 | 4 | 3 | 5 | 3 | 5 | 3 | 5 | 2 | 5 |
| 12 | 3 | NA | NA | 2 | 3 | NA | NA | 4 | 3 |
| 13 | 3 | 3 | 4 | 2 | 3 | 2 | 3 | 2 | 3 |
| 14 | 3 | 2 | 2 | 2 | 2 | 2 | 2 | 2 | 2 |

^R^Additional replicate measurements were taken on different days (numbers in brackets indicate the number of trials on each day)

**Statistical analysis**

**Rejection of speed as a covariate**

Although speed can alter kinematic variables [40], it was decided not to include speed data in the linear mixed models as a covariate for three reasons. First, the speed data could not be aligned to the acceleration data, so stride frequency would have needed to have been used as used an approximation of speed [75]. It is unknown whether stride frequency would be a true reflection of speed or be influenced by other factors, such as training [107, 118], or the shoe and surface effects. For example, stride length has been reported to shorten on soft surfaces [89, 119]. In addition increased fitness could be acquired at a certain times in the year, for example when the racing school runs more jockey courses, and reduce a horse’s stride duration at a given speed [118]. The lack of any clear relationship between the magnitude of CC and DV displacement (Fig 6) would perhaps reflect an ability to alter displacement along these axes independently, with implications for stride duration. Second, calculations of stride frequency in this study are approximate due to variable sample rates of the phones used. Third, estimations of stride frequency over extracted strides indicate the displacement data were collected at 2.10±0.24 Hz and 2.01±0.25 Hz (mean ±2 s.d.) for the horse and rider data, respectively. Given that these values are at or very close to the minimum frequency threshold, the range in stride frequency over the analysed gallop strides must have been low.

**Supplementary Results**

**Overview**

Figures S2 and S3 provide examples of acceleration data in time-series, integrated once to velocity and again to generate the required displacement data for both horse and rider stride cycles, respectively. Figure S5 illustrates the relationship between the magnitude of horse and rider displacements.


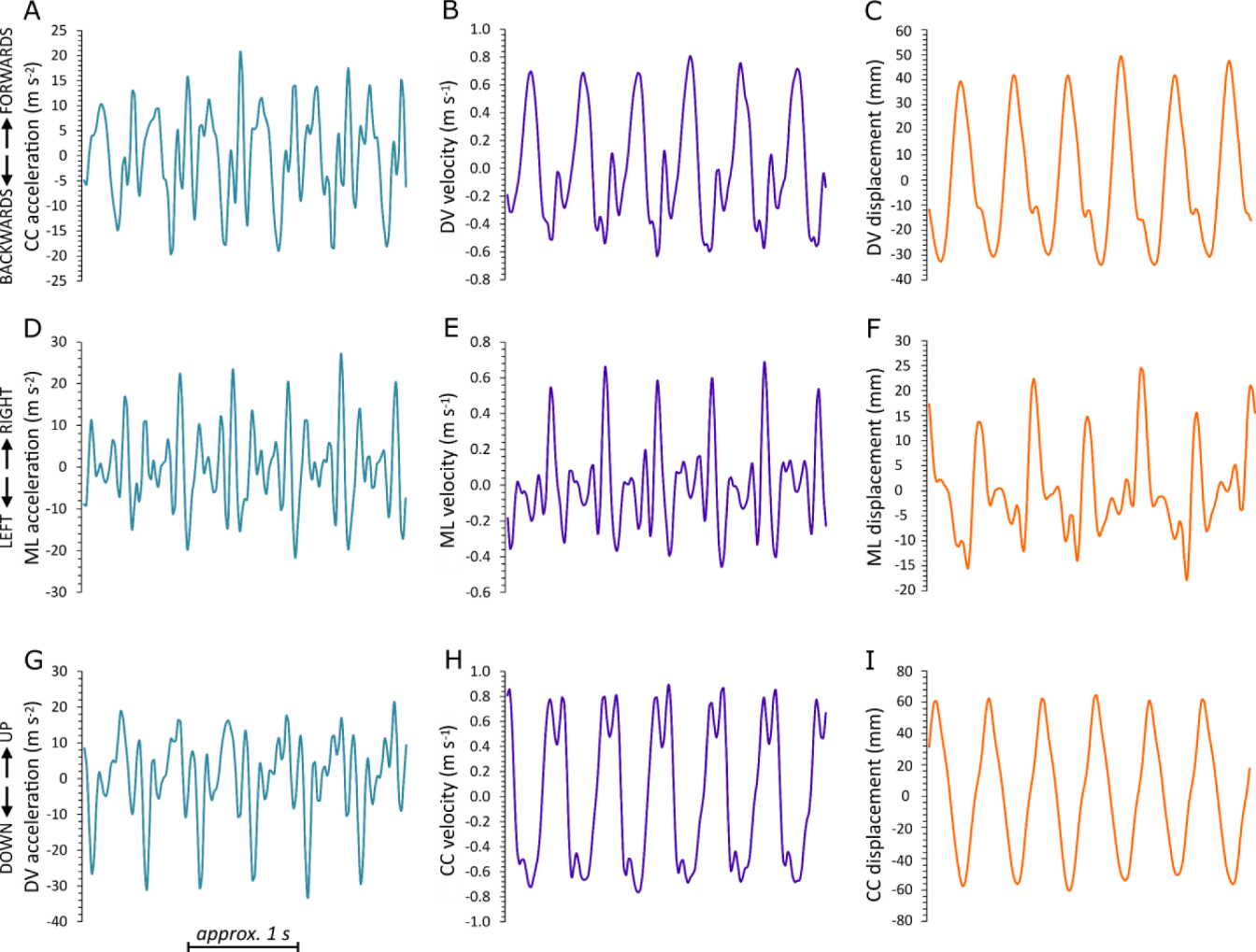


**Fig S2. Examples of filtered acceleration (A, D, G), velocity (B, E, H) and displacement (C, F, I) stride data collected from the iPhone located on the horse’s girth, for cranio-caudal (CC), medio-lateral (ML) and dorso-ventral (DV) axes.** Data were selected at random to represent typical patterns.


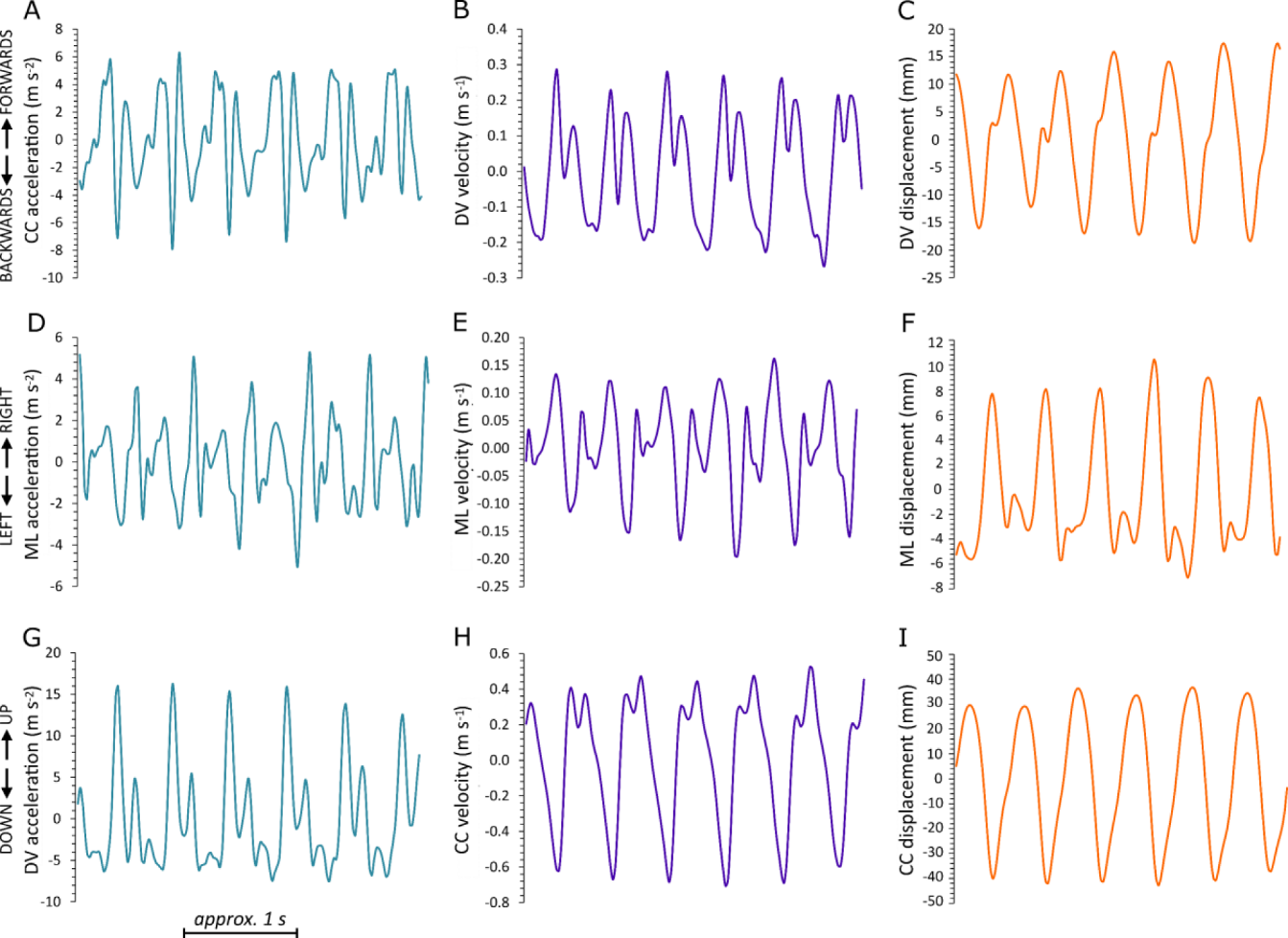


**Fig S3. Example of filtered acceleration (A, D, G), velocity (B, E, H) and displacement (C, F, I) stride data collected from the iPhone located at the rider’s pelvis, for cranio-caudal (CC), medio-lateral (ML) and dorso-ventral (DV) axes.** Data selected correspond with horse data in *Fig S2*.

**Acceleration, velocity and displacement results**

Full details on displacement minima and maxima values for extracted strides used in the mixed model analyses are available in the Supporting Information File S2. Corresponding acceleration and velocity minima and maxima values are also available for reference, but were not the focus of this study. Descriptive statistics (mean, median, standard deviation, standard error, minimum, maximum, range) are also available for each individual data trial.

### Statistical model post-hoc test results

The estimated marginal means and confidence intervals of displacement parameters that were calculated in the linear mixed models are reported in the main text (Tables 3–5) for the surface, shoe and combined shoe-surface effects, respectively. The results of the post-hoc comparisons (with Bonferroni correction) for shoe-type and shoe-surface combinations are presented in Tables S2–S3.

**Table S2. Post-hoc pairwise comparisons (with Bonferroni correction) for shoe type.** Significant differences are highlighted in bold.

| **Displacement parameter** | **Shoe** | **Comparison shoe** | **Significance (Horse)** | **Significance (Rider)** |
| --- | --- | --- | --- | --- |
| CC-axis minima | Aluminium | Barefoot | **<0.0005** | 1.000 |
|  |  | GluShu | **<0.0005** | 1.000 |
|  |  | Steel | **<0.0005** | **<0.0005** |
|  | Barefoot | Aluminium | **<0.0005** | 1.000 |
|  |  | GluShu | 0.128 | 1.000 |
|  |  | Steel | **0.025** | **<0.0005** |
|  | GluShu | Aluminium | **<0.0005** | 1.000 |
|  |  | Barefoot | 0.128 | 1.000 |
|  |  | Steel | 1.000 | **<0.0005** |
|  | Steel | Aluminium | **<0.0005** | **<0.0005** |
|  |  | Barefoot | **0.025** | **<0.0005** |
|  |  | GluShu | 1.000 | **<0.0005** |
| ML-axis minima | Aluminium | Barefoot | na | 1.000 |
|  |  | GluShu | na | 0.192 |
|  |  | Steel | na | **0.005** |
|  | Barefoot | Aluminium | na | 1.000 |
|  |  | GluShu | na | 0.701 |
|  |  | Steel | na | **<0.0005** |
|  | GluShu | Aluminium | na | 0.192 |
|  |  | Barefoot | na | 0.701 |
|  |  | Steel | na | **<0.0005** |
|  | Steel | Aluminium | na | **0.005** |
|  |  | Barefoot | na | **<0.0005** |
|  |  | GluShu | na | **<0.0005** |
| DV-axis minima | Aluminium | Barefoot | 0.138 | 1.000 |
|  |  | GluShu | 0.082 | **<0.0005** |
|  |  | Steel | **<0.0005** | **<0.0005** |
|  | Barefoot | Aluminium | 0.138 | 1.000 |
|  |  | GluShu | **<0.0005** | **<0.0005** |
|  |  | Steel | **<0.0005** | **<0.0005** |
|  | GluShu | Aluminium | 0.082 | **<0.0005** |
|  |  | Barefoot | **<0.0005** | **<0.0005** |
|  |  | Steel | 0.719 | 1.000 |
|  | Steel | Aluminium | **<0.0005** | **<0.0005** |
|  |  | Barefoot | **<0.0005** | **<0.0005** |
|  |  | GluShu | 0.719 | 1.000 |
| CC-axis maxima | Aluminium | Barefoot | **<0.0005** | 1.000 |
|  |  | GluShu | **<0.0005** | 0.061 |
|  |  | Steel | **0.005** | 0.139 |
|  | Barefoot | Aluminium | **<0.0005** | 1.000 |
|  |  | GluShu | 1.000 | 0.188 |
|  |  | Steel | **0.001** | **0.019** |
|  | GluShu | Aluminium | **<0.0005** | 0.061 |
|  |  | Barefoot | 1.000 | 0.188 |
|  |  | Steel | **0.047** | **<0.0005** |
|  | Steel | Aluminium | **0.005** | 0.139 |
|  |  | Barefoot | **0.001** | 0.019 |
|  |  | GluShu | **0.047** | **<0.0005** |
| ML-axis maxima | Aluminium | Barefoot | 1.000 | 0.629 |
|  |  | GluShu | 1.000 | **0.039** |
|  |  | Steel | **0.006** | **<0.0005** |
|  | Barefoot | Aluminium | 1.000 | 0.629 |
|  |  | GluShu | 1.000 | **0.001** |
|  |  | Steel | 0.083 | **<0.0005** |
|  | GluShu | Aluminium | 1.000 | **0.039** |
|  |  | Barefoot | 1.000 | **0.001** |
|  |  | Steel | 0.102 | 0.061 |
|  | Steel | Aluminium | **0.006** | **<0.0005** |
|  |  | Barefoot | 0.083 | **<0.0005** |
|  |  | GluShu | 0.102 | 0.061 |
| DV-axis maxima | Aluminium | Barefoot | 1.000 | **0.038** |
|  |  | GluShu | **<0.0005** | **<0.0005** |
|  |  | Steel | **<0.0005** | **<0.0005** |
|  | Barefoot | Aluminium | 1.000 | **0.038** |
|  |  | GluShu | **<0.0005** | **<0.0005** |
|  |  | Steel | **<0.0005** | **<0.0005** |
|  | GluShu | Aluminium | **<0.0005** | **<0.0005** |
|  |  | Barefoot | **<0.0005** | **<0.0005** |
|  |  | Steel | 1.000 | 1.000 |
|  | Steel | Aluminium | **<0.0005** | **<0.0005** |
|  |  | Barefoot | **<0.0005** | **<0.0005** |
|  |  | GluShu | 1.000 | 1.000 |

**Table S3. Post-hoc pairwise comparisons (with Bonferroni correction) for shoe-surface combinations.** Significant differences are highlighted in bold.

| **Displacement parameter** | **Shoe-surface combination** | **Shoe-surface combination comparison** | **Significance (Horse)** | **Significance (Rider)** |
| --- | --- | --- | --- | --- |
| CC-axis minima |  |  | na | na |
| ML-axis minima minima | Aluminium-Artificial | Aluminium-Turf | **<0.0005** | **<0.0005** |
|  |  | Barefoot_Artificial | 0.066 | **<0.0005** |
|  |  | Barefoot_Turf | **<0.0005** | **<0.0005** |
|  |  | GluShu-Artificial | 1.000 | **<0.0005** |
|  |  | GluShu-Turf | **<0.0005** | **<0.0005** |
|  |  | Steel-Artificial | 1.000 | 1.000 |
|  |  | Steel-Turf | **<0.0005** | **<0.0005** |
|  | Aluminium-Turf | Aluminium-Artificial | **<0.0005** | **<0.0005** |
|  |  | Barefoot_Artificial | **<0.0005** | **<0.0005** |
|  |  | Barefoot_Turf | 1.000 | **<0.0005** |
|  |  | GluShu-Artificial | **<0.0005** | **0.032** |
|  |  | GluShu-Turf | 1.000 | **0.018** |
|  |  | Steel-Artificial | **<0.0005** | **<0.0005** |
|  |  | Steel-Turf | 0.573 | **<0.0005** |
|  | Barefoot_Artificial | Aluminium-Artificial | 0.066 | **<0.0005** |
|  |  | Aluminium-Turf | **<0.0005** | **<0.0005** |
|  |  | Barefoot_Turf | **<0.0005** | 1.000 |
|  |  | GluShu-Artificial | 1.000 | 1.000 |
|  |  | GluShu-Turf | **<0.0005** | 1.000 |
|  |  | Steel-Artificial | 0.635 | **<0.0005** |
|  |  | Steel-Turf | **<0.0005** | 1.000 |
|  | Barefoot_Turf | Aluminium-Artificial | **<0.0005** | **<0.0005** |
|  |  | Aluminium-Turf | 1.000 | **<0.0005** |
|  |  | Barefoot_Artificial | **<0.0005** | 1.000 |
|  |  | GluShu-Artificial | **<0.0005** | 1.000 |
|  |  | GluShu-Turf | 1.000 | 1.000 |
|  |  | Steel-Artificial | **<0.0005** | **<0.0005** |
|  |  | Steel-Turf | **0.024** | 1.000 |
|  | GluShu-Artificial | Aluminium-Artificial | 1.000 | **<0.0005** |
|  |  | Aluminium-Turf | **<0.0005** | **0.032** |
|  |  | Barefoot_Artificial | 1.000 | 1.000 |
|  |  | Barefoot_Turf | **<0.0005** | 1.000 |
|  |  | GluShu-Turf | **<0.0005** | 1.000 |
|  |  | Steel-Artificial | 1.000 | **<0.0005** |
|  |  | Steel-Turf | **<0.0005** | 1.000 |
|  | GluShu-Turf | Aluminium-Artificial | **<0.0005** | **<0.0005** |
|  |  | Aluminium-Turf | 1.000 | **0.018** |
|  |  | Barefoot_Artificial | **<0.0005** | 1.000 |
|  |  | Barefoot_Turf | 1.000 | 1.000 |
|  |  | GluShu-Artificial | **<0.0005** | 1.000 |
|  |  | Steel-Artificial | **<0.0005** | **<0.0005** |
|  |  | Steel-Turf | **0.012** | 1.000 |
|  | Steel-Artificial | Aluminium-Artificial | 1.000 | 1.000 |
|  |  | Aluminium-Turf | **<0.0005** | **<0.0005** |
|  |  | Barefoot_Artificial | 0.635 | **<0.0005** |
|  |  | Barefoot_Turf | **<0.0005** | **<0.0005** |
|  |  | GluShu-Artificial | 1.000 | **<0.0005** |
|  |  | GluShu-Turf | **<0.0005** | **<0.0005** |
|  |  | Steel-Turf | **<0.0005** | **<0.0005** |
|  | Steel-Turf | Aluminium-Artificial | **<0.0005** | **<0.0005** |
|  |  | Aluminium-Turf | 0.573 | **<0.0005** |
|  |  | Barefoot_Artificial | **<0.0005** | 1.000 |
|  |  | Barefoot_Turf | **0.024** | 1.000 |
|  |  | GluShu-Artificial | **<0.0005** | 1.000 |
|  |  | GluShu-Turf | **0.012** | 1.000 |
|  |  | Steel-Artificial | **<0.0005** | **<0.0005** |
| DV-axis minima | Aluminium-artificial | Aluminium-Turf | na | 1.000 |
|  |  | Barefoot-Artificial | na | **0.001** |
|  |  | Barefoot-Turf | na | **0.001** |
|  |  | GluShu-Artificial | na | **<0.0005** |
|  |  | GluShu-Turf | na | **<0.0005** |
|  |  | Steel-Artificial | na | 1.000 |
|  |  | Steel-Turf | na | **<0.0005** |
|  | Aluminium-Turf | Aluminium-artificial | na | 1.000 |
|  |  | Barefoot-Artificial | na | **<0.0005** |
|  |  | Barefoot-Turf | na | 0.052 |
|  |  | GluShu-Artificial | na | **0.009** |
|  |  | GluShu-Turf | na | **<0.0005** |
|  |  | Steel-Artificial | na | 1.000 |
|  |  | Steel-Turf | na | **<0.0005** |
|  | Barefoot-Artificial | Aluminium-artificial | na | **0.001** |
|  |  | Aluminium-Turf | na | **<0.0005** |
|  |  | Barefoot-Turf | na | **<0.0005** |
|  |  | GluShu-Artificial | na | **<0.0005** |
|  |  | GluShu-Turf | na | **<0.0005** |
|  |  | Steel-Artificial | na | **<0.0005** |
|  |  | Steel-Turf | na | **<0.0005** |
|  | Barefoot-Turf | Aluminium-artificial | na | **0.001** |
|  |  | Aluminium-Turf | na | 0.052 |
|  |  | Barefoot-Artificial | na | **<0.0005** |
|  |  | GluShu-Artificial | na | 1.000 |
|  |  | GluShu-Turf | na | 1.000 |
|  |  | Steel-Artificial | na | 0.591 |
|  |  | Steel-Turf | na | **<0.0005** |
|  | GluShu-Artificial | Aluminium-artificial | na | **<0.0005** |
|  |  | Aluminium-Turf | na | **0.009** |
|  |  | Barefoot-Artificial | na | **<0.0005** |
|  |  | Barefoot-Turf | na | 1.000 |
|  |  | GluShu-Turf | na | 1.000 |
|  |  | Steel-Artificial | na | **0.019** |
|  |  | Steel-Turf | na | **<0.0005** |
|  | GluShu-Turf | Aluminium-artificial | na | **<0.0005** |
|  |  | Aluminium-Turf | na | **<0.0005** |
|  |  | Barefoot-Artificial | na | **<0.0005** |
|  |  | Barefoot-Turf | na | 1.000 |
|  |  | GluShu-Artificial | na | 1.000 |
|  |  | Steel-Artificial | na | **0.001** |
|  |  | Steel-Turf | na | **0.002** |
|  | Steel-Artificial | Aluminium-artificial | na | 1.000 |
|  |  | Aluminium-Turf | na | 1.000 |
|  |  | Barefoot-Artificial | na | **<0.0005** |
|  |  | Barefoot-Turf | na | 0.591 |
|  |  | GluShu-Artificial | na | **0.019** |
|  |  | GluShu-Turf | na | **0.001** |
|  |  | Steel-Turf | na | **<0.0005** |
|  | Steel-Turf | Aluminium-artificial | na | **<0.0005** |
|  |  | Aluminium-Turf | na | **<0.0005** |
|  |  | Barefoot-Artificial | na | **<0.0005** |
|  |  | Barefoot-Turf | na | **<0.0005** |
|  |  | GluShu-Artificial | na | **<0.0005** |
|  |  | GluShu-Turf | na | **0.002** |
|  |  | Steel-Artificial | na | **<0.0005** |
| CC-axis maxima | Aluminium-artificial | Aluminium-Turf | na | 1.000 |
|  |  | Barefoot-Artificial | na | 0.928 |
|  |  | Barefoot-Turf | na | 1.000 |
|  |  | GluShu-Artificial | na | 1.000 |
|  |  | GluShu-Turf | na | 1.000 |
|  |  | Steel-Artificial | na | **0.006** |
|  |  | Steel-Turf | na | 1.000 |
|  | Aluminium-Turf | Aluminium-artificial | na | 1.000 |
|  |  | Barefoot-Artificial | na | 1.000 |
|  |  | Barefoot-Turf | na | 0.267 |
|  |  | GluShu-Artificial | na | 1.000 |
|  |  | GluShu-Turf | na | **0.009** |
|  |  | Steel-Artificial | na | 1.000 |
|  |  | Steel-Turf | na | 1.000 |
|  | Barefoot-Artificial | Aluminium-artificial | na | 0.928 |
|  |  | Aluminium-Turf | na | 1.000 |
|  |  | Barefoot-Turf | na | 0.320 |
|  |  | GluShu-Artificial | na | 0.968 |
|  |  | GluShu-Turf | na | **0.009** |
|  |  | Steel-Artificial | na | 1.000 |
|  |  | Steel-Turf | na | 1.000 |
|  | Barefoot-Turf | Aluminium-artificial | na | 1.000 |
|  |  | Aluminium-Turf | na | 0.267 |
|  |  | Barefoot-Artificial | na | 0.320 |
|  |  | GluShu-Artificial | na | 1.000 |
|  |  | GluShu-Turf | na | 1.000 |
|  |  | Steel-Artificial | na | **0.001** |
|  |  | Steel-Turf | na | 0.737 |
|  | GluShu-Artificial | Aluminium-artificial | na | 1.000 |
|  |  | Aluminium-Turf | na | 1.000 |
|  |  | Barefoot-Artificial | na | 0.968 |
|  |  | Barefoot-Turf | na | 1.000 |
|  |  | GluShu-Turf | na | 1.000 |
|  |  | Steel-Artificial | na | **0.005** |
|  |  | Steel-Turf | na | 1.000 |
|  | GluShu-Turf | Aluminium-artificial | na | 1.000 |
|  |  | Aluminium-Turf | na | **0.009** |
|  |  | Barefoot-Artificial | na | **0.009** |
|  |  | Barefoot-Turf | na | 1.000 |
|  |  | GluShu-Artificial | na | 1.000 |
|  |  | Steel-Artificial | na | **<0.0005** |
|  |  | Steel-Turf | na | **0.007** |
|  | Steel-Artificial | Aluminium-artificial | na | **0.006** |
|  |  | Aluminium-Turf | na | 1.000 |
|  |  | Barefoot-Artificial | na | 1.000 |
|  |  | Barefoot-Turf | na | **0.001** |
|  |  | GluShu-Artificial | na | **0.005** |
|  |  | GluShu-Turf | na | **<0.0005** |
|  |  | Steel-Turf | na | 0.560 |
|  | Steel-Turf | Aluminium-artificial | na | 1.000 |
|  |  | Aluminium-Turf | na | 1.000 |
|  |  | Barefoot-Artificial | na | 1.000 |
|  |  | Barefoot-Turf | na | 0.737 |
|  |  | GluShu-Artificial | na | 1.000 |
|  |  | GluShu-Turf | na | **0.007** |
|  |  | Steel-Artificial | na | 0.560 |
| ML-axis maxima | Aluminium-artificial | Aluminium-Turf | **<0.0005** | **<0.0005** |
|  |  | Barefoot-Artificial | 0.463 | **<0.0005** |
|  |  | Barefoot-Turf | **<0.0005** | **<0.0005** |
|  |  | GluShu-Artificial | 1.000 | 0.566 |
|  |  | GluShu-Turf | **<0.0005** | **0.001** |
|  |  | Steel-Artificial | **<0.0005** | 1.000 |
|  |  | Steel-Turf | **<0.0005** | **0.003** |
|  | Aluminium-Turf | Aluminium-artificial | **<0.0005** | **<0.0005** |
|  |  | Barefoot-Artificial | **0.009** | **<0.0005** |
|  |  | Barefoot-Turf | 1.000 | 0.242 |
|  |  | GluShu-Artificial | **<0.0005** | **<0.0005** |
|  |  | GluShu-Turf | 1.000 | **<0.0005** |
|  |  | Steel-Artificial | 1.000 | **<0.0005** |
|  |  | Steel-Turf | 1.000 | **<0.0005** |
|  | Barefoot-Artificial | Aluminium-artificial | 0.463 | **<0.0005** |
|  |  | Aluminium-Turf | **0.009** | **<0.0005** |
|  |  | Barefoot-Turf | **0.045** | **0.003** |
|  |  | GluShu-Artificial | 1.000 | 0.861 |
|  |  | GluShu-Turf | **0.003** | 1.000 |
|  |  | Steel-Artificial | 0.320 | **<0.0005** |
|  |  | Steel-Turf | **0.001** | 1.000 |
|  | Barefoot-Turf | Aluminium-artificial | **<0.0005** | **<0.0005** |
|  |  | Aluminium-Turf | 1.000 | 0.242 |
|  |  | Barefoot-Artificial | **0.045** | **0.003** |
|  |  | GluShu-Artificial | **0.001** | **<0.0005** |
|  |  | GluShu-Turf | 1.000 | **0.001** |
|  |  | Steel-Artificial | 1.000 | **<0.0005** |
|  |  | Steel-Turf | 1.000 | **<0.0005** |
|  | GluShu-Artificial | Aluminium-artificial | 1.000 | 0.566 |
|  |  | Aluminium-Turf | **<0.0005** | **<0.0005** |
|  |  | Barefoot-Artificial | 1.000 | 0.861 |
|  |  | Barefoot-Turf | **0.001** | **<0.0005** |
|  |  | GluShu-Turf | **<0.0005** | 0.639 |
|  |  | Steel-Artificial | **0.034** | **0.017** |
|  |  | Steel-Turf | **<0.0005** | 1.000 |
|  | GluShu-Turf | Aluminium-artificial | **<0.0005** | **0.001** |
|  |  | Aluminium-Turf | 1.000 | **<0.0005** |
|  |  | Barefoot-Artificial | **0.003** | 1.000 |
|  |  | Barefoot-Turf | 1.000 | **0.001** |
|  |  | GluShu-Artificial | **<0.0005** | 0.639 |
|  |  | Steel-Artificial | 1.000 | **<0.0005** |
|  |  | Steel-Turf | 1.000 | 1.000 |
|  | Steel-Artificial | Aluminium-artificial | **<0.0005** | 1.000 |
|  |  | Aluminium-Turf | 1.000 | **<0.0005** |
|  |  | Barefoot-Artificial | 0.320 | **<0.0005** |
|  |  | Barefoot-Turf | 1.000 | **<0.0005** |
|  |  | GluShu-Artificial | **0.034** | 0.017 |
|  |  | GluShu-Turf | 1.000 | **<0.0005** |
|  |  | Steel-Turf | 1.000 | **<0.0005** |
|  | Steel-Turf | Aluminium-artificial | **<0.0005** | **0.003** |
|  |  | Aluminium-Turf | 1.000 | **<0.0005** |
|  |  | Barefoot-Artificial | **0.001** | 1.000 |
|  |  | Barefoot-Turf | 1.000 | **<0.0005** |
|  |  | GluShu-Artificial | **<0.0005** | 1.000 |
|  |  | GluShu-Turf | 1.000 | 1.000 |
|  |  | Steel-Artificial | 1.000 | **<0.0005** |
| DV-axis maxima | Aluminium-artificial | Aluminium-Turf | **<0.0005** | 1.000 |
|  |  | Barefoot-Artificial | **0.003** | 1.000 |
|  |  | Barefoot-Turf | **<0.0005** | **0.002** |
|  |  | GluShu-Artificial | **0.002** | **<0.0005** |
|  |  | GluShu-Turf | **<0.0005** | **<0.0005** |
|  |  | Steel-Artificial | 0.487 | **0.001** |
|  |  | Steel-Turf | **<0.0005** | **<0.0005** |
|  | Aluminium-Turf | Aluminium-artificial | **<0.0005** | 1.000 |
|  |  | Barefoot-Artificial | **<0.0005** | 1.000 |
|  |  | Barefoot-Turf | **0.002** | **<0.0005** |
|  |  | GluShu-Artificial | 1.000 | **<0.0005** |
|  |  | GluShu-Turf | **<0.0005** | **<0.0005** |
|  |  | Steel-Artificial | **0.028** | **0.001** |
|  |  | Steel-Turf | **<0.0005** | **<0.0005** |
|  | Barefoot-Artificial | Aluminium-artificial | **0.003** | 1.000 |
|  |  | Aluminium-Turf | **<0.0005** | 1.000 |
|  |  | Barefoot-Turf | **<0.0005** | **<0.0005** |
|  |  | GluShu-Artificial | **<0.0005** | **<0.0005** |
|  |  | GluShu-Turf | **<0.0005** | **<0.0005** |
|  |  | Steel-Artificial | **<0.0005** | **<0.0005** |
|  |  | Steel-Turf | **<0.0005** | **<0.0005** |
|  | Barefoot-Turf | Aluminium-artificial | **<0.0005** | **0.002** |
|  |  | Aluminium-Turf | **0.002** | **<0.0005** |
|  |  | Barefoot-Artificial | **<0.0005** | **<0.0005** |
|  |  | GluShu-Artificial | **<0.0005** | 1.000 |
|  |  | GluShu-Turf | 1.000 | 0.054 |
|  |  | Steel-Artificial | **<0.0005** | 1.000 |
|  |  | Steel-Turf | **0.056** | **<0.0005** |
|  | GluShu-Artificial | Aluminium-artificial | **0.002** | **<0.0005** |
|  |  | Aluminium-Turf | 1.000 | **<0.0005** |
|  |  | Barefoot-Artificial | **<0.0005** | **<0.0005** |
|  |  | Barefoot-Turf | **<0.0005** | 1.000 |
|  |  | GluShu-Turf | **<0.0005** | 1.000 |
|  |  | Steel-Artificial | 1.000 | 1.000 |
|  |  | Steel-Turf | **<0.0005** | **<0.0005** |
|  | GluShu-Turf | Aluminium-artificial | **<0.0005** | **<0.0005** |
|  |  | Aluminium-Turf | **<0.0005** | **<0.0005** |
|  |  | Barefoot-Artificial | **<0.0005** | **<0.0005** |
|  |  | Barefoot-Turf | 1.000 | 0.054 |
|  |  | GluShu-Artificial | **<0.0005** | 1.000 |
|  |  | Steel-Artificial | **<0.0005** | **0.045** |
|  |  | Steel-Turf | 1.000 | **0.029** |
|  | Steel-Artificial | Aluminium-artificial | 0.487 | **0.001** |
|  |  | Aluminium-Turf | **0.028** | **0.001** |
|  |  | Barefoot-Artificial | **<0.0005** | **<0.0005** |
|  |  | Barefoot-Turf | **<0.0005** | 1.000 |
|  |  | GluShu-Artificial | 1.000 | 1.000 |
|  |  | GluShu-Turf | **<0.0005** | **0.045** |
|  |  | Steel-Turf | **<0.0005** | **<0.0005** |
|  | Steel-Turf | Aluminium-artificial | **<0.0005** | **<0.0005** |
|  |  | Aluminium-Turf | **<0.0005** | **<0.0005** |
|  |  | Barefoot-Artificial | **<0.0005** | **<0.0005** |
|  |  | Barefoot-Turf | 0.056 | **<0.0005** |
|  |  | GluShu-Artificial | **<0.0005** | **<0.0005** |
|  |  | GluShu-Turf | 1.000 | **0.029** |
|  |  | Steel-Artificial | **<0.0005** | **<0.0005** |

#

# Additional References

117. Google (2020) *The British Racing School.* Available at: http://maps.google.co.uk (Accessed: 12 January 2020).

118. Parkes RSV, Weller R, Pfau T, Witte TH. The effect of training on stride duration in a cohort of two-year-old and three-year-old thoroughbred racehorses. Animals. 2019;9: 1–11. doi:10.3390/ani9070466

119. Chateau H, Holden L, Robin D, Falala S, Pourcelot P, Estoup P, et al. Biomechanical analysis of hoof landing and stride parameters in harness trotter horses running on different tracks of a sand beach (from wet to dry) and on an asphalt road. Equine Vet J. 2010;42: 488–495. doi:10.1111/j.2042-3306.2010.00277.x
